# Perception of Patients with Cancer Enquiring About Adjuvant Therapy with Cannabis Medicine for Palliation of Symptoms: An Interview Study among Danish Health Care Professionals

**DOI:** 10.1089/pmr.2021.0056

**Published:** 2022-04-26

**Authors:** Dorte Buchwald, Kristina A.I Winter, Dorte Brønnum, Dorte Melgaard, Peter D.C. Leutscher

**Affiliations:** ^1^ Unit of Palliative Care, North Denmark Regional Hospital, Hjoerring, Denmark.; ^2^ Center for Clinical Research, North Denmark Regional Hospital, Hjoerring, Denmark.; ^3^ Department of Clinical Medicine, Aalborg University, Aalborg, Denmark.

**Keywords:** cancer, cannabinoids, cannabis, health care professionals, interview survey, palliative care

## Abstract

**Background:**

A medicinal cannabis pilot program was launched in Denmark in 2018 to support patients as countermeasure against self-medication by use of cannabis products from the illicit market. The aim was to facilitate patient access to adjuvate therapy using medicinal cannabis under the guidance of physicians.

**Objective:**

The aim of this interview study was to elucidate how health care professionals (HCPs) perceive cancer patients enquiring about cannabis medicine (CM), including medicinal cannabis and cannabis-based medicine, for adjuvant palliative therapy.

**Design:**

The program used semistructured qualitative research interviews with thematic analysis.

**Setting/Participants:**

Fifty HCPs took part in the study with 10 informants in each of the following 5 groups: oncologists, palliative care specialists, general practitioners, registered nurses in oncology, and in palliative care.

**Results:**

The informants reported that optional CM as adjuvant therapy was only discussed when initiated by the patient or relatives. Reluctance by HCPs to enter into a dialogue about CM with their patients was mainly explained by the lack of clinical evidence for the use of CM in palliative care of patients with cancer. None of the oncologists had ever prescribed CM, while three palliative care specialists and two general practitioners had issued prescriptions on rare occasions.

**Conclusion:**

HCPs involved in cancer treatment and palliative care are in general reluctant to discuss optional adjuvant CM therapy with their patients. The Danish health care authorities need to address this barrier to ensure that patients eligible for CM therapy are given this option as intended by the launch of the national pilot program.

## Introduction

Patients with advanced cancer often suffer from a complex pattern of symptoms related to the cancer disease itself and adverse reactions to treatment.^1–3^ Conventional drug regimens for palliation of pain, including opioids, anticonvulsants or tricyclic antidepressants, and other symptom alleviating agents, may not always achieve adequate effect or may cause intolerable side effects, such as headache, dizziness, nausea, and constipation adding to the overall burden of symptoms in the patients, hence contributing to further deterioration of quality of life.

In recent years, there has been an increasing interest among patients and informal caregivers, in use of cannabis medicine (CM) for palliative care of cancer-related symptoms.^4–8^ In this given context, the term CM covers medicinal cannabis and cannabis-based medicine, respectively, although a distinction should be made between medicinal cannabis and cannabis-based medicine regarding origin and content of active molecules, including cannabinoids, terpenoids, and isoflavones. In Denmark, patients and physicians commonly refer to medicinal cannabis and cannabis-based medicine without having a fundamental knowledge regarding the characteristics of each category.

The majority of research literature related to cancer patients receiving CM originates from prospective, nonrandomized, unblinded observational studies,^9–14^ and just a few randomized controlled trials (RCTs).^15–18^ However, the published RCTs have predominantly been focusing on pain as the only symptom to evaluate upon regarding efficacy and safety of CM, whereas other essential symptoms have not been subject for similar investigation. Thus, further evidence-based clinical research is lacking to determine the role of CM therapy in palliative care.^6–7,19–21^ This mission is today met by different obstacles mainly due to constrained access to funding. In Denmark, conduction of RCTs has also been challenged by extremely long and complicated procedures for approval of CM products by the Danish Medicines Agency.

In 2018, a four-year program focusing on use of medicinal cannabis was launched in Denmark. The political intention of the pilot program was to ensure patients' access to the Danish Medicines Agency certified medicinal cannabis (MC) products prescribed by a physician. In 2019, an interview study was conducted in a group of 20 Danish patients with advanced cancer and a history of CM use. The study showed that patients were mainly using medicinal cannabis of illicit origin. Moreover, the patients reported a preference of CM products being prescribed by a physician as opposed to a patient self-therapeutic approach.^22^ However, the patients reported that they found it difficult to reach the health care professionals (HCPs) to discuss optional CM therapy.^22,23^ The aim of the study was to explore perceptions among different subgroups of HCPs providing care to patients with cancer about adjuvant CM therapy for palliation of symptoms.

## Methods

Semistructured qualitative research interviews were conducted in 5 groups with 10 HCPs in each group. The interviews took place from February to May 2019.

The 50 HCPs were recruited from 2 of 5 Danish regions, North Denmark Region (*n* = 44), and Central Denmark Region (*n* = 6), respectively. The HCPs in the five group were randomly selected oncology physicians and oncology nurses, providing care to curative as well as terminal cancer patients, palliative care physicians and palliative care nurses providing care to terminal cancer patients, and primary care physicians involved in investigation of cancer being suspected and providing supportive care to cancer patients in general.

Written or verbal informed consent was obtained before the interviews. Signed consent forms, audio files, and transcripts from interviews were stored in accordance with the General Data Protection Regulation. A nurse PhD in palliative care conducted the interviews. Anonymity was ensured and data were handled confidentially. Face-to face interviews were audio recorded and transcribed verbatim. Four of the interviews with oncology physicians were conducted by telephone.

An interview guide was used covering three main themes concerning CM therapy: Dialogues, dilemmas, and prescription practices. As Danish nurses cannot prescribe CM, they were not asked questions about prescription practice. The term CM was used systematically in the interviews.

The interviews were analyzed within the interdisciplinary research group from a thematic perspective,^24^ and interpretations were continuously challenged and discussed. First, the transcribed material was read several times to identify central statements and phrases to the themes studied. Second, quotations were identified representing thematically relevant responses by the informants.^25^

The North Denmark Region Committee on Health Research Ethics issued a waiver regarding need of their approval being an consent-based interview study.

## Results

The findings covering the three interview themes (dialogues, dilemmas and prescription practices) are presented below.

### Dialogues

All groups of informants stated that they did not actively initiate a dialogue with patients about optional CM therapy. Oncology physicians expressed that they felt reluctant to discuss CM with their patients due to the lack of RCTs. They tended only to ask about CM if the patients showed symptoms interpreted as potential adverse effects caused by CM, “*It is not something we talk about as a routine unless the patient has raised liver enzymes.*” Oncology nurses occasionally had conversations with a patient following the scheduled consultation with the oncologist, “*We tend to feel that if we reject a discussion about cannabis, we also reject the patient. At least, this is how we think the patient may feel.*”

Palliative care physicians were willing to engage in a dialogue about CM therapy, but only at the request of the patient or an informal caregiver. Their main purpose of having a dialogue was to maintain trust. Palliative care nurses experienced that patients seemed anxious to talk about CM, but they felt constrained in discussing the topic due to the lack of RCTs. Primary care physicians felt that they were not able to advice patients on this issue because they lacked knowledge of the clinical use of CM, especially regarding dosing and drug interactions.

### Dilemmas

The interviews revealed different dilemmas. Oncology physicians felt that the Danish MC pilot program had been pushed forward by politicians, patient organizations, and private stakeholders, “*There is a new movement towards public negligence when it comes to medical expertise, which is no longer respected.*” Palliative care physicians were concerned because the Danish Medicines Agency had placed the clinical responsibility of CM therapy on physicians. Moreover, palliative care physicians experienced that primary care physicians expected them to prescribe CM, “*We do not want to be a cannabis prescription factory with general practitioners referring cancer patients to us*.”

Primary care physicians expressed frustration that the Danish Medicines Agency had placed the clinical responsibility of CM therapy on physicians without communication with the health professionals' organizations. Oncology nurses felt trapped between physicians and patients concerning CM issues. Palliative care nurses expressed a dilemma when they had contact with patients they considered potential candidate for CM therapy for the relief of symptoms, but were unable to assist pursuit of this option due to a conflicting policy.

### Prescription practices

In Denmark, physicians may prescribe CM according to the mission statement of the MC pilot program. None of the oncology physicians had ever prescribed CM. Three palliative care specialists and two primary care physicians had prescribed CM on rare occasions. Oncology physicians were not willing to prescribe CM on principle. Oncology physicians expressed understanding for patients who expressed hope that CM could have a curative effect on their cancer but would not prescribe CM from this point of view, “*To give cannabis as an indication and hope for curing cancer makes no sense to me as a physician.*” Three palliative care physicians were willing to prescribe CM as a last option when other medications were not adequately relieving symptoms.

Most of the palliative care physicians were reluctant to prescribe CM for relief of symptoms, but found it important to be respectful toward the patient when they explained their reasons for not prescribing CM due to worries about side effects “*I feel a great responsibility. If there were any side effects, I would be the one to blame.*” Primary care physicians advocated that prescribing CM to patients was outside their role, “*I do not prescribe cannabis. I would refer the patient to the palliative team.*”

## Discussion

There is a barrier between patients and clinicians as lack of evidence has been highlighted as a major barrier for prescribing CM products by oncology physicians participating in a questionnaire survey in Minnesota.^26^ However, two-thirds of the oncology participants in the Minnesota study supported the integration of optional CM regimens in oncology care; none of the Danish oncology physicians participating in our interview study supported this view. Physicians participating in similar surveys have reported that their knowledge and experience with CM therapy is generally too limited to discuss the therapeutic options with their patients.^26–28^

An additional dilemma in the context of using CM in patients with advanced cancer receiving palliative care is that, in addition to seeking symptom relief, many of these patients carry a hope for a potentially curative effect of CM.^22,29^ The two different treatment scopes may well create an ambivalent situation for the HCPs to meet the expectations of the patients. Although this situation may conflict with the professional integrity of the HCPs, they may decide to focus on the palliative care element of CM therapy exclusively as the one and only clinical reason for a prescription.

It is important that HCPs engage proactively in the dialogue with patients about CM. Policies are required to ensure that physicians and patients have access, in protocolized manner, to information about CM treatment options. However, a patient may not necessarily obtain a prescription for CM in the setting. That will, in the end, be in the discretion of the individual physician to make this decision based upon medical practice preferences and clinical comfort.

The qualitative method was chosen as we found this best suited to a study focusing on understanding phenomena that cannot be quantified and reduced to operational values. A limitation of the study was that some interviews were conducted by telephone. One of the study's strengths was that we included a relatively large number of participants to be interviewed covering a broader composition of professional background. Furthermore, that the final analyzed interviews did not contribute to new critical insights and allow us to conclude that the theoretical saturation was reached.^30^

## Conclusion

The HCPs in this interview study were generally reluctant to discuss adjuvant CM therapy with their patients due to the lack of evidence for use of CM. A few of the palliative care physicians and primary care physicians had prescribed CM on rare occasions; none of the oncology physicians had ever prescribed CM. The HCPs expressed different dilemmas such as how the clinical responsibility of CM therapy was placed on physicians without a treatment guideline having been made available for them. These different issues need to be addressed to meet the patients in their search for dialogue and support around MC.

## Abbreviations Used

CM: cannabis medicine
HCPs: health care professionals
MC: medicinal cannabis
RCTs: randomized controlled trials

## References

[1] Portenoy RK, Thaler HT, Kornblith AB, et al.: Symptom prevalence, characteristics and distress in a cancer population. Qual Life Res 1994;3:183–189. 10.1007/BF00435383 7920492

[2] Teunissen SC, Wesker W, Kruitwagen C, et al.: Symptom prevalence in patients with incurable cancer: A systematic review. J Pain Symptom Manage 2007;34:94–104. 10.1016/j.jpainsymman.2006.10.015 17509812

[3] Van Lancker A, Velghe A, Van Hecke A, et al.: Prevalence of symptoms in older cancer patients receiving palliative care: A systematic review and meta-analysis. J Pain Symptom Manage 2014;47:90–104. 10.1016/j.jpainsymman.2013.02.016 23764109

[4] Abrams DI, and Guzman M: Cannabis in cancer care. Clin Pharmacol Ther 2015;97:575–586 10.1002/cpt.108 25777363

[5] MacCallum CA, and Russo EB: Practical considerations on medical cannabis administration and dosing. European J Int Med 2018;49:12–19. 10.1016/j.ejim.2018.01.004 29307505

[6] National Academies of Sciences, Engineering and Medicine: The Health Effect of Cannabis and Cannabinoids: The Current State Evidence and Recommendations for Research. The National Academies Press, 2017. 28182367

[7] Health Cannada: *Information for Health Care Professionals. Cannabis (Marihuana, Marijuana) and the Cannabinoids*. Ministry of Health, 2018.

[8] Kleckner AS, Kleckner IR, Kamen CS, et al.: Opportunities for cannabis in supportive care in cancer. Adv Med Oncol 2019;11:1–29. 10.1177/1758835919866362 PMC6676264 31413731

[9] Maida V, Ennis M, Irani S, et al.: Adjunctive nabilone in cancer pain and symptom management: A prospective observational study using propensity scoring. J Support Oncol 2008;6:119–124. 18402303

[10] Bar-Sela G, Vorobeichik M, Drawsheh S, et al.: The medical necessity for medicinal cannabis: Prospective, observational study evaluating the treatment in cancer patients on supportive or palliative care. Evid Based Complement Alternat Med 2013;2013:510392. 23956774 10.1155/2013/510392 PMC3730175

[11] Waissengrin B, Urban D, Leshem Y, et al.: Patterns of use of medical cannabis among Israeli cancer patients: A single institution experience. J Pain Symptom Manage 2015;49:223–230. 10.1016/j.jpainsymman.2014.05.018 24937161

[12] Bar-Lev Schleider L, Mechoulam R, Lederman V, et al.: Prospective analysis of safety and efficacy of medical cannabis in large unselected population of patients with cancer. Eur J Intern Med 2018;49:37–43. 10.1016/j.ejim.2018.01.023 29482741

[13] Anderson SP, Zylla DM, and McGriff DM, Arneson T: Impact of Medical Cannabis on Patient-Reported symptoms for patients with cancer enrolled in Minnesota's Medical Cannabis Program. J Oncol Pract 2019;15:e338–e345. 10.1200/JOP.18.00562 30860938

[14] Good PD, Greer RM, Huggett GE, and Hardy JR: An open-label pilot study testing the feasibility of assessing total symptom burden in trials of cannabinoid medications in palliative care. J Palliat Med 2020;23:650–655. 10.1089/jpm.2019.0540 PMC7232640 31800354

[15] Johnson JR, Burnell-Nugent M, Lossignol D, et al.: Multicenter, double-blind, randomized, placebo-controlled, parallel-group study of the efficacy, safety, and tolerability of THC:CBD extract and THC extract in patients with intractable cancer-related pain. J Pain Symptom Manage 2010;39:167–179. 10.1016/j.jpainsymman.2009.06.008 19896326

[16] Brisbois TD, de Kock IH, Watanabe SM, et al.: Delta-9-tetrahydrocannabinol may palliate altered chemosensory perception in cancer patients: Results of a randomized, double-blind, placebo-controlled pilot trial. Ann Oncol 2011;22:2086–2093 10.1093/annonc/mdq727 21343383

[17] Portenoy RK, Ganae-Motan ED, Allende S, et al.: Nabiximols for opioid-treated cancer patients with poorly controlled chronic pain: A randomized placebo-controlled. Graded-dose trial. J Pain 2012;13:438–449. 10.1016/j.jpain.2012.01.003 22483680

[18] Fallon M, Lux EA, McQuade R, et al.: Sativex oromucosal spray as adjunctive therapy in advanced cancer patients with chronic pain unalleviated by optimized opioid therapy: Two double-blind, randomized, placebo-controlled phase 3 studies. Br J Pain 2017;11:119–133. 10.1177/2049463717710042 PMC5521351 28785408

[19] Whiting PF, Wolff RF, Deshpande S, et al.: Cannabinoids for medical use: A systematic review and meta-analysis. JAMA 2015;313:2456–2473. 10.1001/jama.2015.6358 26103030

[20] Häuser W, Fitzcharles MA, Radbruch L, and Petzke F: Cannabinoids in pain management and palliative medicine. Dtsch Arztebl Int 2017;114:627–634. 10.3238/arztebl.2017.0627 PMC5645627 29017688

[21] Mücke M, Weier M, Carter C, et al.: Systematic review and meta-analysis of cannabinoids in palliative medicine. J Cachexia Sarcopenia Muscle 2018;9:220–234. 10.1002/jcsm.12273 PMC5879974 29400010

[22] Buchwald D, Brønnum D, Melgaard D, and Leutscher PDC: Living with a hope of survival is challenged by a lack of clinical evidence: An interview study among cancer patients using cannabis-based Medicine. J Palliat Med 2019;23:1090–1093 10.1089/jpm.2019.0298 31710262

[23] Cyr C, Arboleda MF, Aggarwal SK, et al.: Cannabis in palliative care: Current challenges and practical recommendations. Ann Palliat Med 2018;7:463–477. 10.21037/apm.2018.06.04 30180728

[24] Kvale S, and Brinkmann S: Interviews: Learning the Craft of Qualitative Research Interviewing, 3rd ed. SAGE Publications Inc., 2014.

[25] Manen MV: *Researching Lived Experience. Human Science for an Action Sensitive Pedagogy, 2nd ed*. LONDON, Ont: Althouse Press, 1997.

[26] Zylla D, Steele G, Eklund J, et al.: Oncology clinicians and the Minnesota medical cannabis program: A survey on medical cannabis practice patterns, barriers to enrollment, and educational needs. Cannabis Cannabinoid Res 2018;3:195–202. 10.1089/can.2018.0029 PMC6225592 30426072

[27] Karanges EA, Suraev A, Elias N, et al.: Knowledge and attitudes of Australian general practitioners towards medicinal cannabis: A cross-sectional survey. BMJ Open 2018;8:e022101. 10.1136/bmjopen-2018-022101 PMC6042562 29970456

[28] Braun IM, Wright A, Peteet J, et al.: Medical oncologists' beliefs, practices, and knowledge regarding marijuana used therapeutically: A nationally representative survey study. J Clin Oncol 2018;36:1957–1962. 10.1200/JCO.2017.76.1221 PMC6553839 29746226

[29] Zarrabi AJ, Welsh JW, Sniecinski, et al.: Perception of benefits and harms of medical cannabis among seriously ill patients in an outpatient palliative care practice. J Palliat Med 2020;23:558–562. 10.1089/jpm.2019.0211 31539298

[30] Delmar C: “Generalizability” as recognition: Reflections on a foundational problem in qualitative research. Qual Stud 2010;1:115–128. DOI: 10.7146/qs.v1i2.3828.

